# The Inferior Grain Filling Initiation Promotes the Source Strength of Rice Leaves

**DOI:** 10.1186/s12284-023-00656-x

**Published:** 2023-09-16

**Authors:** Zhengrong Jiang, Hongyi Yang, Meichen Zhu, Longmei Wu, Feiyu Yan, Haoyu Qian, Wenjun He, Dun Liu, Hong Chen, Lin Chen, Yanfeng Ding, Soulaiman Sakr, Ganghua Li

**Affiliations:** 1 Sanya Institute of Nanjing Agriculture, Jiangsu Collaborative Innovation Center for Modern Crop Production, Key Laboratory of Crop Physiology Ecology and Production Management, Nanjing Agricultural University, Sanya, 572000 China; 2 China- Kenya Belt and Road Joint Laboratory on Crop Molecular Biology, Nanjing, 210095 China; 3 Institut Agro, University of Angers, INRAE, IRHS, SFR 4207 QUASAV, Angers, 49000 France; 4 Rice Research Institute, Guangdong Academy of Agricultural Sciences, Guangzhou, 510640 China; 5 School of Life Sciences and Food Engineering, Huaiyin Institute of Technology, Huai’an, 223003 China

**Keywords:** Rice, Sink, Source, Sugar, Phytohormone

## Abstract

Poor grain-filling initiation in inferior spikelets severely impedes rice yield improvement, while photo-assimilates from source leaves can greatly stimulate the initiation of inferior grain-filling (sink). To investigate the underlying mechanism of source-sink interaction, a two-year field experiment was conducted in 2019 and 2020 using two large-panicle rice cultivars (CJ03 and W1844). The treatments included intact panicles and partial spikelet removal. These two cultivars showed no significant difference in the number of spikelets per panicle. However, after removing spikelet, W1844 showed higher promotion on 1000-grain weight and seed-setting rate than CJ03, particularly for inferior spikelets. The reason was that the better sink activity of W1844 led to a more effective initiation of inferior grain-filling compared to CJ03. The inferior grain weight of CJ03 and W1844 did not show a significant increase until 8 days poster anthesis (DPA), which follows a similar pattern to the accumulation of photo-assimilates in leaves. After removing spikelets, the source leaves of W1844 exhibited lower photosynthetic inhibition compared to CJ03, as well as stronger metabolism and transport of photo-assimilates. Although T6P levels remained constant in both cultivars under same conditions, the source leaves of W1844 showed notable downregulation of SnRK1 activity and upregulation of phytohormones (such as abscisic acid, cytokinins, and auxin) after removing spikelets. Hence, the high sink strength of inferior spikelets plays a role in triggering the enhancement of source strength in rice leaves, thereby fulfilling grain-filling initiation demands.

**Supplementary Information:**

The online version contains supplementary material available at 10.1186/s12284-023-00656-x.

## Introduction

Rice (*Oryza sativa* L.) acts a staple role in food production for entire global population. However, to meet the escalating demands, rice production needs to approximately double by 2030 (Foley et al. 2011). In China, high-yield rice cultivars, particularly large-panicle rice, have gained significant importance due to their potential for increased yield with a large number of spikelets (You et al. 2016). Nevertheless, in large-panicle rice, the filling of inferior spikelets (IS) on the secondary branches of the panicle often lags behind the superior spikelets (SS) on the primary branches after flowering, thereby limiting the yield (Fu et al. 2011; Chen et al. 2019). Inferior grain-filling is mainly limited by poor initiation, which is associated with poor sink activity (enzyme activity of grains to utilize carbohydrates) during grain filling initiation (Chen et al. 2019; Jiang et al. 2021). The initiation of inferior grain filling is influenced by various factors, including sink strength (sink size and sink activity), source strength (capacity to supply photo-assimilates), and flow strength (capacity to transport carbohydrates) (Deng et al. 2021). Photosynthesis of source leaves is responsible for producing carbohydrates to meet the demand of sink growth in rice (Makino 2011; Zhu et al. 2022). Fully mature source leaves have the capacity to export up to 80% of photo-assimilates to sink tissues (Kalt-Torres and Huber 1987). Since the allocation of carbohydrates between sink and source is crucial determinant of crop yield (Braun et al. 2014), maintaining a balance in the partitioning of photo-assimilates from source to sink is essential for the initiation of grain filling in rice.

The cytoplasm of source mature leaves synthesized sucrose through the photosynthetic conversion of carbon dioxide to triose phosphate during the daytime, and starch remobilization occurs at night (Wang et al. 2021a, b). The main carbohydrate transported in rice is sucrose, which is transported into the apoplast space by OsSWEET11 (*Oryza sativa* Sugar Will Eventually Be exported Transporter 11) before being actively loaded in the leaf phloem by OsSUTs (*Oryza sativa* Sucrose transporters) against a concentration gradient (Hu et al. 2021; Wang et al. 2021a, b). Sucrose-phosphate synthase (SPS), encoded by *OsSPS1*, is responsible for generating sucrose by photosynthesis (Gesch et al. 2002; Ohara et al. 2010). The genes of *OsSUS3* and *OsSUS4* encode sucrose synthase (SuSase), initiating the first degradative step of sucrose utilization (Yao et al. 2019). The *OsAGPL1* gene encodes the ADP-glucose pyrophosphorylase (AGPase) to regulate starch synthesis, while the α-Amylase (*OsAmy3*) catalyzes starch degradation (Meng et al. 2020). These processes are crucial for providing carbon and energy to prevent sugar starvation (Graf and Smith 2011). However, the heavy carbohydrate accumulation of leaves can repress photosynthesis and regulate sugar distribution in plants (Goldschmidt and Huber 1992; Ainsworth and Bush 2011). Several studies have shown that sink strength plays a crucial role for controlling plants growth and the rate of photosynthetic activity in source leaves (Sonnewald and Fernie 2018; Xu et al. 2021; Cabon et al. 2022; Dai et al. 2023). The activities associated with high sink growth need to consider not only the efficiency of photo-assimilate production and sucrose transport, but also the sink's capacity to utilize carbohydrates (Chen et al. 2019; Jiang et al. 2021). However, the underlying relationship between sink strength and source strength remains unclear.

Source activities, including photosynthesis activity, are typically regulated by photo-assimilate allocation and phytohormones metabolism (Rolland et al. 2006; Yu et al. 2015; Müller and Munné-Bosch 2021). Recently, the sugar signaling pathway involving trehalose-6-phosphate (T6P) and Snf1-related protein kinase-1 (SnRK1) has gained considerable attention due to their sensitiveness to allocation of photo-assimilate and phytohormones (Jiang et al. 2021; Ishihara et al. 2022). The T6P level, serving as an indicator of sucrose availability, is synthesized by trehalose-6-phosphate synthase (TPS) and degraded by trehalsoe-6-phosphate phosphatase (TPP), which significantly stimulates starch biosynthesis in leaves through post-translational regulation of AGPase (Martins et al. 2013; Ishihara et al. 2022). The SnRK1 signaling pathway, acting as the core regulator of carbon and energy sensing in various plant organelles (Tsai and Gazzarrini 2014), is inhibited by sugars and closely intertwined with phytohormone signaling pathways (Hulsmans et al. 2016; Baena-González and Hanson 2017). However, the roles of sugar signaling and hormone levels in the source-sink interaction have thus far investigated separately and in different biological contexts.

As a major regulator for photosynthesis and abiotic stress, the phytohormone abscisic acid (ABA) can repress photo-assimilates production of source leaf (Pantin et al. 2012). The ABA accumulation of leaves favors the stomatal closure (Kim et al. 2010) and downregulation of several genes involved in photosynthesis (Zhu et al. 2020). The genes *OsNCED1* and *OsABA3* can regulate ABA biosynthetic pathway in rice leaves (Zeng et al. 2015; Zhang et al. 2021; Liu et al. 2022; Zhou et al. 2022), while the expression of *OsCYP707A6* can promote ABA degradation (Piao et al. 2019). Intriguingly, cytokinin can antagonize the inhibitory effects of ABA by optimizing photosynthetic efficiency in leaves (Müller and Munné-Bosch 2021; Wang et al. 2021a, b). Cytokinins (CKs), such as zeatin (ZT), have a pivotal role in affecting both the functional and structural aspects of photosynthesis machinery (Hönig et al. 2018; Mao et al. 2022). Meanwhile, CKs control carbohydrate transport by regulating SWEETs and SUTs transporters, influencing the source-sink interaction (McIntyre et al. 2021). In addition, the auxin (IAA) content has a positive correlation with photosynthesis activity in some species, indicating a regulatory role in chloroplast development and stomata patterning (Tivendale and Millar 2022). Notebly, ABA, CKs, and IAA are important for photo-assimilates remobilization and the sugar signaling of SnRK1 pathway (Yu et al. 2015). All these findings underline the crucial role of hormone in regulating photosynthesis and carbon level in source leaves (Yu et al. 2015).

Here, we examined the relationship of source-sink by comparing their roles in the same study model. Two large-panicle rice varieties (CJ03 and W1844), with different sink strength in inferior spikelets, provide an excellent system to study the relationship between leaf source strength and grain filling initiation. In a two-year field experiment, we aimed to test the following hypotheses: (1) The initiation of inferior grain filling drives source leaves photosynthesis. (2) High sink strength promotes carbohydrate transport and allocation in source leaves during grain filling initiation. (3) A sugar and hormones-dependent mechanism is involved in the regulation of sink strength on source leaves.

## Materials and Methods

### Plant Materials and Management

Field experiments were conducted in a randomized block design with three replications in 2019 and 2020, at Danyang Experimental Base of Nanjing Agricultural University, Jiangsu Province, China (31°54′31″ N, 119′28′21″ E). The size of plot was 7 m × 10 m. According to our previous data (Jiang et al. 2021), two homozygous large-panicle rice cultivars, CJ03 and W1844, were selected to analyze the deep relationship of sink strength and source leaves during grain filling. These two cultivars were coming from the State Key Laboratory of Rice Genetics and Germplasm Innovation, Nanjing Agricultural University. Seeds were sown on May 21 in 2019 and May 23 in 2020. The 25-d old seedlings were transplanted with two seedlings per hill, at a hill spacing of 13.3 cm × 30 cm. The soil of experimental site was clay loam. The basic physical and chemical properties of 0 ~ 20 cm soil in 2019 and 2020 are showed in Additional file 1: Table S1. A total of 280 kg ha^−1^ nitrogen (N) was applied with the ratio of 5:5 at one day before transplanting and the day of leaf-age remainder 3.5, respectively. The meteorological data, which were measured during the growth period of CJ03 and W1844 at a weather station in the experimental site, are shown in Additional file 1: Fig. S1. All agronomic management practices (e.g., pest, weed control, and water management) were all done following local recommendations.

The time (50% panicles emerged from flag-leaf sheath) was recorded as heading date of each rice cultivar. The time (98% grains in the field turned yellow) was recorded as maturity date of each rice cultivar. Detail information of two rice cultivars was shown in Additional file 1: Table S2 (e.g., the heading and maturity dates, duration from heading to maturity, and the total growth duration).

### Experimental Design

A total of 1400 panicles with similar growth patterns that headed on the same day were chosen and labeled from each plot. The flowering date and the position of each spikelet on the tagged panicles were recorded. The spikelet-thinning treatment was performed according to our previous protocol on the flowering date of CJ03 and W1844 (Jiang et al. 2021). In general, the experiment included two treatment groups: the control group with no spikelet thinning (labeled as T0 group) and the upper 2/3 followers removed (labeled as T1 group) when the lower-part inferior spikelets were flowering (Additional file 1: Fig. S2). The difference of flowering date between upper-part superior spikelets (SS) and lower-part inferior spikelets (IS) was nearly 4–5 days in a panicle. In this experiment, SS was the grain locating in the first three primary branches of the upper part of the panicle, and IS was the grain locating in the last three second branches in the basal part (Jiang et al. 2021).

### Sampling and Analysis

#### Agronomic Analysis of Panicle

All the panicles were harvested at maturity in 2019 and 2020. Agronomic features were measured, and then dried at 80 °C for 1 week. Dry weights of grains were measured to calculated 1000-grain weight. Seed setting rate was calculated by using Kobata’s method (Kobata et al. 2013). Superior spikelets (SG) rate and inferior spikelets (IG) rate on per panicle were calculated as follows.

Seed setting rate = plump grain number/total grain number

SG rate = superior spikelets located on all primary branches /total grain number

IG rate = inferior spikelets located on all secondary branches of rice panicle/total grain number

#### Grain Weight

The grain weight of superior spikelets and inferior spikelets were measured at early grain filling stage and maturity in 2019 and 2020. According to our previous method (Jiang et al. 2021), we sampled about 30 tagged panicles from each replicate plot every 2 days post anthesis (DPA) during early grain-filling stage (2, 4, 6, 8, and 10 DPA). Additionally, about 30 panicles from each replicated plot were sampled at maturity. The panicle of T0 group (no spikelets thinning) were separated into two parts: SS (all superior spikelets on the upper three primary branches of panicle), IS (all inferior spikelets on the basal three secondary branches of panicle). And the panicle of T1 group (upper 2/3 spikelets removed) were separated into one part: IS (all inferior spikelets on the basal three secondary branches of panicle). All the samples were dried in the oven at 105 °C for 30 min, and dried at 80 °C for 1 week.

#### Analysis of Photosynthesis

On 8 DPA of inferior spikelets, the photosynthesis of the flag leaves was measured with a portable photosynthesis system (LI-6400; Li-Cor) at 10 am. On sunny day, the measurement was conducted in 9 tagged rice in each replicate plot with a constant saturated light level of 1500 μmol m^−2^ s^−1^ provided by red/blue light-emitting diodes. Leaf temperature was maintained at 30 °C and relative humidity in the chamber at c. 0–6 (humidity deficit c. 1.1 kPa). Before using gas-exchange measurements for data analysis, the flag leaves were allowed to equilibrate nearly 20 min at each setting.

#### Analysis of Dry Weight and Carbohydrates Accumulation in Leaves

For dry weight analysis, all the source leaves of 9 tagged plants were sampled from each replicate plot on 8 DPA of inferior spikelets. The leaves were first dried in the oven at 105 °C for 30 min, and then dried at 80 °C for 1 week before getting dry weight of source leaves.

All the upper three leaves of 30 tagged panicles were sampled from each replicate plot on 8 DPA of inferior spikelets. The leaves were first dried in the oven at 105 °C for 30 min, and then dried at 80 °C for 1 week. The samples were ground to fine powder and weighed nearly 100 mg per replicant for sucrose and starch extraction. The sucrose and starch extraction methods were flowed according to Cock et al. (1976). Final values were expressed as mg g^−1^ dry weight for comparison. For sucrose analysis, the reaction mixture was measured at 480 nm. The starch analysis needs to be done after extracting sucrose, the reaction mixture was read at 620 nm.

#### Analysis of Key Enzyme Activities in Leaves

On 8 DPA of inferior spikelets, the upper three leaves of 30 tagged plants were sampled from each replicant in the morning. These samples were used to determine the enzymes activities (SPS, AGPase, α-Amylase, and SnRK1). The leaves were first frozen in liquid nitrogen for 1 min before storing at − 80 °C, and ground to fine powder. Before adding 1 mL extraction buffer, all the samples were weighed about 100 mg fine powder for testing enzyme activity. The activity of SPS was determined using the method described by Okamura et al. (2011). AGPase activity was measured based on the method outlined by Nakamura et al. (1989). α-amylase activity was evaluated using the method developed by Bhatia and Singh. (2002). The SnRK1 activity was measured according to the method of Samuel Bledsoe et al. (2017) and determined as described procedure Zhang et al. (2009). The samples were measured following the instructions.

#### Detection of Trehalose-6-phosphate and Hormone Level

On 8 DPA of inferior spikelets, about 30 tagged panicles were sampled their upper three leaves from each replicant for detection of trehalose-6-phosphate and hormone level. The samples were first frozen in liquid nitrogen for 1 min before storing at − 80 °C. All the samples were ground to fine powder for next step.

For trehalose-6-phosphate (T6P) assay, the analysis way was according to our previous method (Jiang et al. 2021). The T6P of the purified plant was used to capture the antibody and coat the microplate to make a solid-phase antibody. The samples were added into the coated microplate in turn and combined with Horse Radish Peroxidase (HRP) labeled detection antibody to form antibody-antigen enzyme-labeled antibody complex. Then it is important to add tetramethylbenzidine (TMB) for developing the color after thorough washing. TMB is trans-formed into blue under the catalysis of the HRP enzyme and yellow under the action of acid. For T6P content measurement, the supernatant was measured at 450 nm before calculating by standard curve.

For analysis of hormone content, the method was slightly modified according to Fang et al. (2018). About 250 mg samples were extracted with 1 mL pre-cooled methanol/water/formic acid (15:4:1, v/v/v). The mixture was kept in dark at 4 °C for overnight. Then the mixture was centrifuged at 12,000 rpm for 15 min at 4 °C, repeating three times to collect all the supernatants. The nitrogen gas stream was used to evaporate the combined extracts to dryness. And then the dryness was reconstituted in 80% (v/v) methanol before filtering with C18 columns (Waters, Sep-Pak ® Vac, 6 cc, 500 mg) at 4 °C. The extracts were analyzed using high-performance liquid chromatography-tandem mass spectrometry (HPLC–MS/MS) analysis. In this system, the mobile phase contained methanol and ultrapure water containing 0.5% formic acid. With a flow rate of 0.25 mL min^–1^, 5 μL of each sample was injected into a ZORBAX SB-C18 (Agilent Technologies) column (2.1 mm × 150 mm; 3.5 mm) to test the content of ABA, IAA and ZT. MS conditions were as follows: the pressure of the air curtain, nebulizer, and aux gas were 15, 65, and 70 psi, respectively; and the atomizing temperature was 400 °C, the spray voltage was 4500 V.

#### Analysis of Relative Genes Expression

The upper three leaves of 15 tagged panicles were sampled from each replicant on 8 DPA of inferior spikelets. The leaves were first frozen in liquid nitrogen for 1 min before storing at minus 80 °C. Before RNA extraction, the samples were ground to fine powder and weighed nearly 100 mg per replicant for next step. Total RNA was extracted by using Plant RNA Kit (Omega Biotek, Inc., USA), and reversed-transcribed into the first-strand cDNA with the Prime-Script-TM RT Reagent Kit (Takara, Kyoto, Japan), oligo-dT. The qRT-PCR was performed by an ABI 7300 and SYBR Premix Ex Taq-TM (Takara, Kyoto, Japan) according to the manufacturer's protocol. All the experiments were analyzed by three biological replicants with three technical repeats per biological replicate. As shown in Additional file 1: Table S3, the cDNA was amplified by specific primers of 5′-UTR and 3′-UTR for the analysis of relative gene expression (*OsSWEET11*, *OsSUT1*, *OsSUT2*, *OsSUT4*, *OsSPS1*, *OsSUS3*, *OsSUS4*, *OsAGPL1*, *OsAmy3*, *OsTPS1*, *OsTPP2*, *OsTPP6, OSK1*, *OSK24*, *OSK35*, *OsNCED1*, *OsABA3*, and *OsCYP707A6*).

### Statistical Analysis

Statistics were evaluated by analysis of variance (ANOVA). For all data, *P* < 0.05 was considered statistical significance. Statistical analyses were performed using SPSS Statistics (IBM SPSS Inc, Chicago, IL, USA).

## Results

### Pattern of Grain Dry Matter Accumulation

**Fig. 1:**
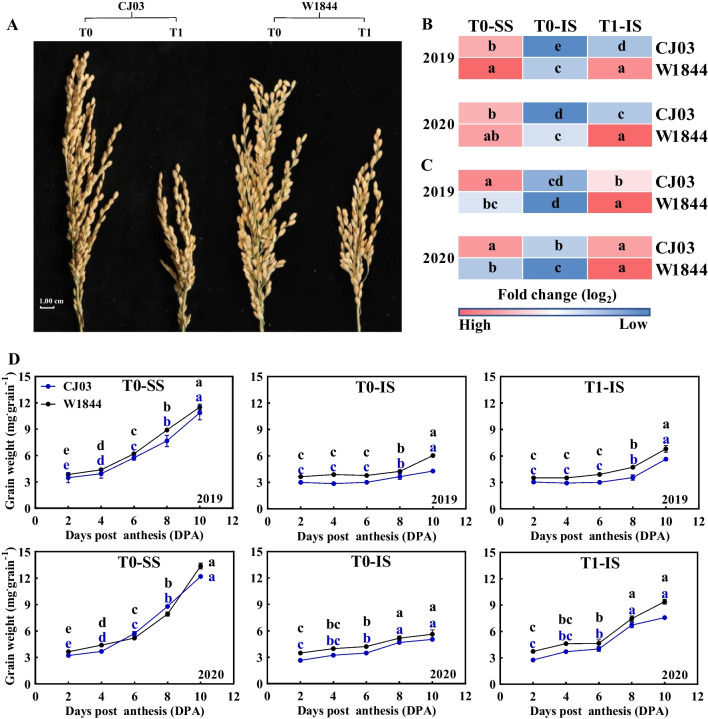
Changes in grain growth during grain filling stage in 2019 and 2020. **A** schematic diagram of rice panicle in CJ03 and W1844 at maturity; **B** heat maps of grain weight in different position at maturity; **C** heat maps of seed setting rate in different position at maturity; **D** dynamic of grain weight in CJ03 and W1844 during early grain filling stage; T0, control group with no removing-spikelets; T1, removing top 2/3 of the spikelets in panicle; SS, superior spikelets; IS, inferior spikelets; Significant differences at each time point with same color are indicated by different letters (*P* < 0.05) as determined by Duncan’s test; The data are the means of three replications ± SD (n = 3)

At maturity, there was no significant difference in the number of spikelets per panicle between the T0 group of CJ03 and W1844 in both 2019 and 2020 (Table 1). The values were approximately 267.67 and 259.75 for CJ03, and 275.67 and 273.67 for W1844. A large number of inferior spikelets were located on the secondary branches of rice panicle of CJ03 and W1844, while W1844 had a higher rate of inferior spikelets compared to CJ03 (Table 1, Fig. 1A). Intriguingly, the seed setting rate of W1844 in T0 group was notably lower than that of CJ03 in 2019 and 2020 (Table 1), while the 1000-grain weight of W1844 (22.91 g and 24.35 g) was significantly higher than that of CJ03 (21.87 g and 22.38 g). After removing superior spikelets, the seed setting rate of both CJ03 and W1844 increased significantly. In 2019 and 2020, the 1000-grain weight of W1844 (25.95 g and 25.19 g) remained significantly higher than that of CJ03 (20.10 g and 20.97 g) (Table 1).

**Table 1:** Agronomic traits of test materials at maturity in 2019 and 2020

Year	Variety	Treatment	Spikelets per panicle	1000-grain weight (g)	Seed setting rate (%)	
2019	CJ03	T0	267.67a	21.87c	87.67c	
		T1	91.17b	20.10d	93.00b	
	W1844	T0	275.67a	22.91b	84.92d	
		T1	97.92b	25.95a	95.58a	
2020	CJ03	T0	259.75a	22.38c	92.25b	
		T1	77.50c	20.97d	94.17a	
	W1844	T0	273.67a	24.35b	85.17c	
		T1	92.58b	25.19a	94.67a	

Year	Variety	Treatment	SG per panicle	IG per panicle	SG rate (%)	IG rate (%)
2019	CJ03	T0	104.65a	163.02b	39.10a	60.90b
		T1	31.04c	60.13d	34.05a	65.95b
	W1844	T0	68.31b	207.36a	24.78b	75.22a
		T1	24.15d	73.77c	24.67b	75.33a
2020	CJ03	T0	80.83a	178.92b	31.12a	68.88b
		T1	23.25c	54.25d	30.00a	70.00b
	W1844	T0	67.33b	206.34a	24.60b	75.40a
		T1	22.42c	70.16c	24.21b	75.79a

T0, control group with no removing-spikelets; T1, removing top 2/3 of the spikelets in panicle; SG, superior spikelets located on all primary branches of rice panicle; IG, inferior spikelets located on all secondary branches of rice panicle; Different letters indicate statistically significant differences under the same year at the *P* = 0.05 level; The data are the means of three replications ± SD, consisting of 30 plants each

In 2019 and 2020, the spikelets removal led to a significant improvement in both the dry grain-weight and seed setting rate of inferior spikelets (Fig. 1). Notably, the inferior grain weight of W1844 T1 group could even surpass the weight of superior spikelets in T0 group, while CJ03 did not exhibit this characteristic (Fig. 1B). Meanwhile, the seed setting rate of IS in T1 group was significantly higher than that of SS in W1844 (Fig. 1C). Based on the observed long stagnation in accumulation of inferior grain weight (Fig. 1D), the grain weight of IS in CJ03 and W1844 could not be significantly increased until 8 DPA. However, the accumulation of inferior grain in W1844 was higher than that of CJ03 during the early gran filling stage (Fig. 1D and Additional file 1: Fig. S3), which may be attributed to the higher sink strength in the inferior spikelets of W1844, as suggested by previous studies (Jiang et al. 2021).

### Difference of Photosynthesis in Leaves

**Fig. 2:**
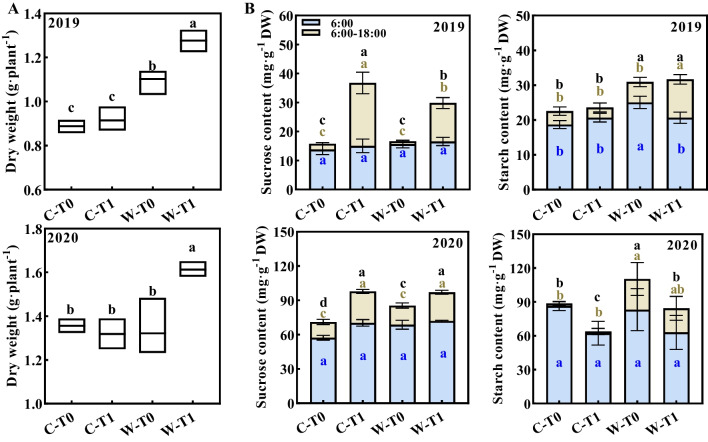
The accumulation of carbohydrates in source leaves of CJ03 and W1844 in 2019 and 2020. **A** Dry weight of total source leaves in CJ03 and W1844 at 8 DPA; **B** photosynthetic accumulation of top three leaves in CJ03 and W1844 at 8 DPA; C, CJ03; W, W1844; T0, control group with no removing-spikelets; T1, removing top 2/3 of the spikelets in panicle; DPA, days post anthesis; Significant differences are indicated by different letters with same color (*P* < 0.05) as determined by Duncan’s test. Bars mean SD (n = 9)

From 8 DPA onwards, along with the initiation of inferior grain filling, the accumulation of photo-assimilate (sucrose and starch) in CJ03 and W1844 exhibited a significantly increase (Fig. 1D and Additional file 1: Figs. S3-S4). To assess the difference in photosynthesis in the source leaves of CJ03 and W1844, measurements were taken at 8 DPA in 2019 and 2020 (Table 2, Fig. 2). No notable differences in photosynthetic parameters were observed between the T0 group of CJ03 and W1844 (Table 2). However, in 2019, the dry weight of source leaves in W1844 was significantly higher than that of CJ03 (Fig. 2A). After removing spikelets, the photosynthetic parameters of flag leaves were decreased in both varieties (Table 2), but those of W1844 remained higher than that of CJ03. The dry weight of source leaves in W1844 noticeably increased after removing spiklets, which was significantly higher than that of CJ03 (Fig. 2A). The accumulation of sucrose in leaves of both CJ03 and W1844 exhibited an increasing trend during the daytime of 8 DPA, while those of W1844 notably exhibited higher starch accumulation than CJ03 (Fig. 2B). After removing spikelets, only the leaves of W1844 (W-T1 group) had a significant increase in starch accumulation, compared with CJ03 (C-T1 group) (Fig. 2B). Although the daily accumulation of starch content did not exhibit significant changes after removing spikelets in 2020 for both CJ03 and W1844, the sucrose accumulation in the source leaves clearly increased in both 2019 and 2020 (Fig. 2B). This difference might be attributed to the variation in source strength between CJ03 and W1844.

**Table 2:** Differential sensitivity of photosynthesis in flag leaves to remove spikelets in CJ03 and W1844 at 8 DPA in 2019 and 2020

Year	Variety	Treatment	Net photosynthetic rate (umol·m^−2^ s^−1^)	Stomatal conductance (mmol·m^−2^ s^−1^)	Intercellular CO_2_ concentration (μmol·mol^−1^)	Trmmol rate (mmol·m^−2^ s^−1^)
2019	CJ03	T0	22.51a	0.65b	285.30b	6.53a
		T1	20.06c	0.52c	268.55c	5.32c
	W1844	T0	21.91a	0.74a	305.32a	6.28a
		T1	20.72b	0.60bc	274.15b	5.68b
2020	CJ03	T0	25.40a	0.86a	225.50ab	13.73a
		T1	20.92c	0.54c	210.57c	11.72c
	W1844	T0	24.91a	0.89a	233.63a	13.78a
		T1	21.92b	0.75b	220.72b	12.71b

T0, control group with no removing-spikelets; T1, removing top 2/3 of the spikelets in panicle; Different letters indicate statistically significant differences under the same year at the *P* = 0.05 level; The data are the means of three replications ± SD, consisting of 9 plants each

### Sucrose Loading and Carbon Metabolism in Leaves

**Fig. 3:**
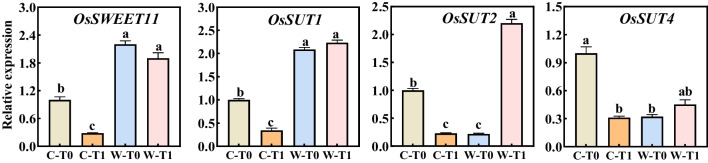
Relative expression levels of sucrose transporters in the top three leaves at 8 DPA in 2020. Significant differences are indicated by different letters (*P* < 0.05) as determined by Duncan’s test; The data are the means of three biological replications ± SD, consisting of 3 technical replications in each biological replication

The difference in sucrose loading of the leaves were examined between CJ03 and W1844 at 8 DPA (Fig. 3), by investigating the transcript levels of four main sucrose transporters (*OsSWEET11*, *OsSUT1*, *OsSUT2*, and *OsSUT4*). The gene expression of *OsSWEET11* and *OsSUT1* in leaves of W1844 was significantly higher than that of CJ03 (Fig. 3). After removing spikelets, the gene expression of *OsSWEET11* and *OsSUTs* (*OsSUT1*, *OsSUT2*, and *OsSUT4*) significantly decreased in leaves of CJ03, while W1844 did not show any repression in the expression of sucrose transporters. Compared to W-T0 group, the expression level of *OsSUT2* is doubled in leaves of W1844 (W-T1), while the expression levels of *OsSUT1* and *OsSUT4* remain unchanged (Fig. 3). These results emphasize the high sucrose loading ability of W1844 source leaves in response to the removal of spikelets, supporting findings from previous studies (Chen et al. 2019).

**Fig. 4:**
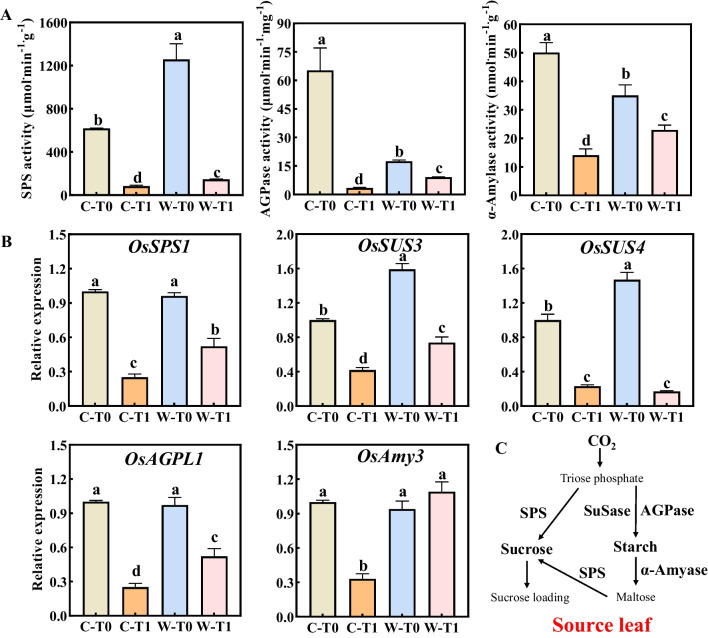
Activities of key enzymes and relative gene expression on carbon metabolism in the top three leaves at early grain filling stage of 2020. **A** The activity of SPS, AGPase, and α-Amylase in the top three leaves at 8 DPA; **B** the level of gene expression relating carbon metabolism in the top three leaves at 8 DPA; **C** working model of carbon metabolism in source leaves of rice during daytime; C, CJ03; W, W1844; T0, control group with no removing-spikelets; T1, removing top 2/3 of the spikelets in panicle; Four types of genes expression level were measured: relating sucrose synthesis (*OsSPS1*), relating starch synthesis (*OsSUS3*, *OsSUS4*, and *OsAGPL1*), and relating starch degradation (*OsAmy3*); The data are the means of three biological replications ± SD, consisting of 3 technical replications in each biological replication

To investigate the potential link between sucrose-loading ability and carbon metabolism in source leaves, we evaluated the key enzymes activities (SPS, AGPase, and α-Amylase) and gene expression (*OsSPS1*, *OsSUS3*, *OsSUS4*, *OsAGPL1*, and *OsAmy3*) related to sucrose-starch conversion (Fig. 4). Comparing with same treatment, the SPS activity in leaves of W1844 was obviously higher than that of CJ03 (Fig. 4A). The removal of spikelets resulted in a huge decrease in starch metabolizing enzymes (AGPase and α-Amylase) in the leaves of both CJ03 and W1844. However, the leaves of W1844 T1 group exhibited significantly higher activities in these enzymes compared to CJ03. Most of the gene expression (*OsSPS1*, *OsSUS3*, *OsSUS4*, *OsAGPL1*) related to sucrose-starch conversion decreased significantly after removing spikelets, particularly for source leaves of CJ03 (Fig. 4B).

### Sugar Signaling and Hormones Content

**Fig. 5:**
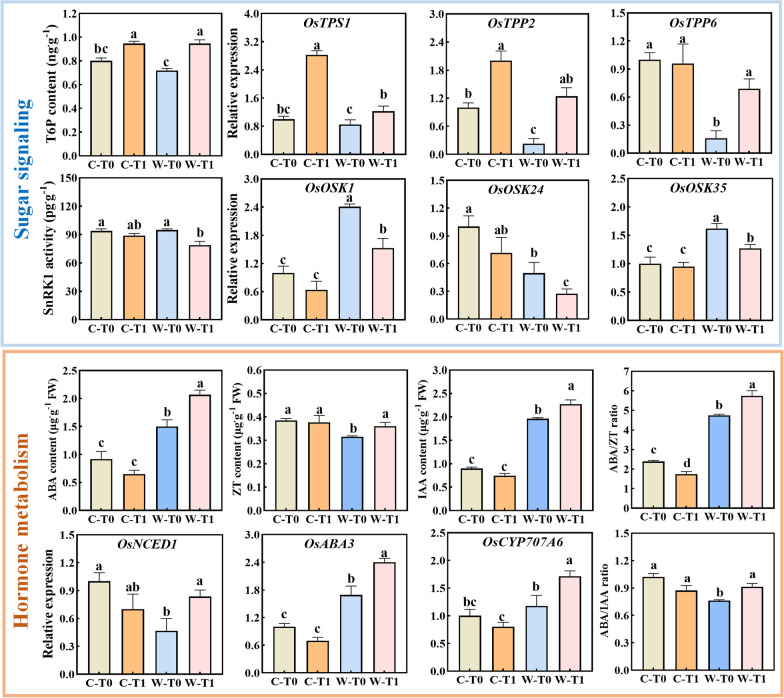
Antagonistic regulation of compound levels and gene expression levels relating to the hormone-sugar pathway in the top three leaves at 8 DPA of 2020. C, CJ03; W, W1844; T0, control group with no removing-spikelets; T1, removing top 2/3 of the spikelets in panicle; Blue color correspond to sugar signaling pathway; Yellow color correspond to hormone metabolism; Different letters indicate significant differences among treatments (*P* < 0.05); The data are the means of three biological replications ± SD, consisting of 3 technical replications in each biological replication

The compound levels and gene expression levels relating to the module T6P/SnRK1 signaling and to some hormones (CKs, IAA and ABA) were tested in source leaves at 8 DPA (Fig. 5). After removing spikelets (groups of C-T1 and W-T1), the T6P content significantly increased in CJ03 and W1844, which is in accordance with the upregulation of TPS (*OsTPS1*). The expression of *OsTPP2* was also increased in the same conditions while that of *OsTPP6* were only notably upregulated in W-T1 group. SnRK1 is subdivided into two subgroups, SnRK1a (*OSK1*) and SnRK1b (*OSK24* and *OSK35*), acting to counterbalance the level of T6P and maintain an appropriate level of sucrose in plant (Tsai and Gazzarrini 2014; Figueroa and Lunn 2016). After removing spikelets, the SnRK1 activity and relative gene expression of *OsOSK1*, *OsOSK24*, and *OsOSK35* were down-regulated in W1844 (W-T1 group). However, there was no significant difference in different treatments of CJ03. Meanwhile, only the leaves of W1844 showed a significant up-regulation in hormone content in both photosynthesis-repressing phytohormones (ABA) and photosynthesis-promoting phytohormones (ZT, IAA). In addition, the expression of genes for ABA-synthesizing enzymes (*OsNCED1* and *OsABA3*) and ABA-catabolizing enzyme (*OsCYP707A6*) were significantly up-regulated in leaves of W1844 after removing spikelets. The elevated ABA content in leaf of W1844 T1 group would result from-a homeostasis of ABA biosynthesis and degradation.

## Discussion

### The Photosynthesis Is Promoted by the Initiation of Inferior Grain Filling

The poor sink strength of inferior spikelets in rice cultivars could result in poor grain filling (Liang et al. 2001; Kato 2020; Jiang et al. 2021). The rate of inferior grains in entire panicle (IG rate) was high in both CJ03 and W1844, and the IG rate of W1844 was significantly higher than that of CJ03 in 2019 and 2020 (Table 1). Thus, the poor grain filling of inferior spikelets could hugely limit the yield of CJ03 and W1844. The seed setting rate of CJ03 and W1844 both increased significantly after removing superior spikelets, but the dry grain-weight and seed setting rate of inferior spikelets in W1844 could showed greater improvement compared to CJ03 in 2019 and 2020 (Fig. 1). Available studies have found that the long lag phase and poor initiation of inferior grain filling were the major limitation to poor grain filling of inferior spikelets (Das et al. 2016; Chen et al. 2019). The initiation of inferior grain filling began at 8 DPA in both CJ03 and W1844, with higher grain weight accumulation observed in W1844 IS compared to CJ03 (Fig. 1D). This higher sink accumulation potentially resulted by higher sink activity in the inferior spikelets of W1844 (Fig. 1). Improving source supply has become an important way to promote inferior grain filling and crop yield (Fu et al. 2011; Won et al. 2022; Slafer et al. 2023). Thus, it is necessary to deeply understand the relationship of source supply and inferior grain growth.

The photo-assimilates accumulation of source leaves in CJ03 and W1844 did not increased significantly until 8 DPA (Additional file 1: Fig. S3), which was similarly to the trend of grain weight accumulation during grain filling initiation in both varieties (Fig. 1D and Additional file 1: Fig. S3). Spikelets removal resulted in a reduction of photosynthetic parameters in the flag leaves, while the W1844 T1 group exhibited higher photosynthetic parameters and dry weight in the source leaves (Table 2; Fig. 2A). The inferior spikelets of W1844 exhibited higher sink activity compared to CJ03 (Jiang et al. 2021), as evidenced by a superior capacity for grain-filling initiation (Fig. 1). The pervasive control of sinks over plant growth and carbon partitioning becomes increasingly prominent (Smith et al. 2018; Bairam et al. 2019; Cabon et al. 2022). Defoliation of source leaves leads to an increase in the photosynthetic rate of the remaining leaves to match the rate photo-assimilates use in sinks (McIntyre et al. 2021). These results indicated that there was a potential regulation between the initiation of grain filling of inferior grains and the photosynthesis of source leaves. Less sink demand represses the sucrose export from source leaves (Slafer et al. 2023), leading to elevated level of sucrose in source leaves in CJ03 and W1844 after removing spikelets (Fig. 2B). The source leaves of W1844 showed enhanced daytime starch accumulation compared to CJ03 at 8 DPA, especially in 2020, indicating its stronger source strength during grain filling initiation, particularly after spikelet removal (Fig. 2B). Therefore, the high sink strength of inferior spikelets in W1844 promote source strength during grain filling initiation.

### High Sink Strength Triggers Sucrose Loading and Carbon Metabolism in Source Leaves During Inferior Grain-Filling Initiation

Photosynthesis of source leaves is a key step in producing carbohydrates to meet the demand of highly metabolic active in sink (Jansson et al. 2018). At 8 DPA, when the initiation of inferior grain filling began (Fig. 1D and Additional file 1: Fig. S3), the accumulation of photo-assimilates (sucrose and starch) significantly increased in source leaves (Fig. 1 and Additional file 1: Fig. S4). Meanwhile, a number of genes and enzymes related to sucrose-loading and sucrose-starch conversion came into play in source leaves (Figs. 3 and 4).

Sucrose loading is the initial process of transporting sucrose from source (leaves) to sink (grains) and is essential for increasing rice yields (Rennie and Turgeon 2009; Wang et al. 2015). The expression of rice sucrose transporters *OsSWEET11* and *OsSUT1* in sources leaves of W1844 was two-fold higher than of CJ03 (Fig. 3). Spikelets removal resulted lower expression of *OsSWEET11* and *OsSUT1* in source leaves of CJ03, while the expression of W-T1 group remains five-fold higher than C-T1 group (Fig. 3). The rice sucrose transporters, *OsSUT2* and *OsSUT4*, are strongly associated with the accumulation of soluble carbohydrates and the photosynthetic maintenance (Mengzhu et al. 2020; Zhang et al. 2020; Wang et al. 2021a, b). Notably, the expression of *OsSUT2* and *OsSUT4* in W1844 was not significantly down-regulated by removing spikelets, and the expression of *OsSUT2* in source leaves of W1844 T1 group was higher than that of CJ03 (Fig. 3). According to our findings and previous researches (McCormick et al. 2009; Chen et al. 2019), the high sucrose-loading ability of W1844 T1 group could be possibly due to W1844's high sink strength, resulting from its strong sink demand and sucrose import. It was observed that a high source accumulation, achieved through spikelet removal, resulted in significant sucrose accumulation in source leaves. This photosynthetic production exceeds the sink demand, providing why some carbohydrates are allocated to starch accumulation and the stimulation of leaf biomass. It will be interesting to study how sink strength affects all these processes.

The key carbohydrate metabolic enzymes (*eg*. SPS, AGPase, and α-Amyase) play a crucial role in regulating metabolic status of source leaves to balance source-sink dynamics (Mathan et al. 2021). The α-Amylase (*OsAmy3*) is known as the major enzyme for starch degradation, which can hydrolyze starch into sucrose for use and export carbon (Zhao et al. 2021). Removing spikelets significantly decreased these activities and relative gene expressions, indicating that the conversion of sucrose and starch were reduced in rice leaves, particularly for CJ03 (Fig. 4). These data revealed that sink size was a crucial factor for regulating photosynthesis and sucrose metabolism in source leaves. Intriguingly, the SPS activity and expression of *OsSPS1* in W1844 T1 group were significantly higher than that of CJ03, along with higher activities of AGPase and α-Amylase and relative genes expression (Fig. 4). The higher levels of carbon metabolism and sucrose loading in the source leaves, as well as the differential expression patterns of related genes, suggest that the sink of the W1844 T1 group receives a greater amount of sucrose utilization from the source leaves as compared to CJ03. The promotion of photosynthesis and sucrose-loading in source leaves may be attributed to the high sink strength of inferior spikelets in W1844.

### The Regulation of Sink Strength on Source Strength May Involve a Sugar and Hormones-Dependent Mechanism

The sugar signaling of T6P-SnRK1 pathway is intimately linked to photosynthesis of source leaves, and is primarily involved in carbohydrate and energy metabolism, stress responses, and plants growth (Czedik-Eysenberg et al. 2016; Wurzinger et al. 2018; Baena-Gonzalez and Lunn 2020). T6P concentration is sensitive to the changes of sugar abundance (Jiang et al. 2022), Spikelets removal led to an increased accumulation of sucrose and promoted T6P metabolism in the source leaves of both CJ03 and W1844 (Figs. 2 and 5). Moreover, SnRK1 functions as a central sensor of sugar status and abiotic stress, enabling plants to properly balance sugar production and consumption for growth (Lin et al. 2014; Nukarinen et al. 2016). Removing spikelets noticeable down-regulated the SnRK1 signaling pathway in source leaves of W1844, while CJ03 showed no significant difference (Fig. 5). SnRK1 signaling, as the central mediator, undergoes constant changes in response to the carbon demand of the sink (Wurzinger et al. 2018). It is activated by energy deprivation and hormone signals while being inactivated by carbohydrates that restore energy balance in source and sink organs (Baena-González et al. 2007; Mair et al. 2015). Spikelet removal inhibited photosynthesis in CJ03 and W1844, with higher accumulation in the W1844 T1 group attributed to its strong sink and source characteristics (Table 2; Fig. 2). Compared to W1844 T0 group, the high sugar status of sources leaves in W1844 T1 group could contribute to downregulation of SnRK1 in source leaf. The poor inferior spikelet initiation in CJ03 lowered sink carbon demand and sucrose loading in source leaves (Figs. 1 and 3). Conversely, the high sink strength of W1844 inferior spikelets activated the central sugar signaling pathway of SnRK1, enhancing grain filling initiation.

**Fig. 6:**
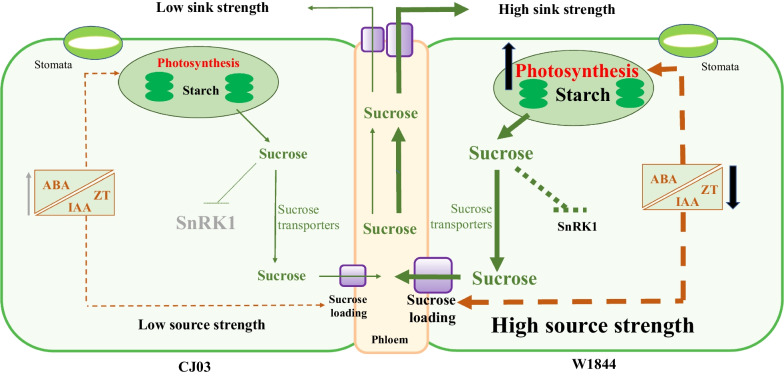
A working model of rice leaves in response to altered sink–source relations at early grain filling stage

The response of sugar levels and hormone signaling is closely linked to photosynthesis to response changing environmental cues (Jossier et al. 2009; Rodrigues et al. 2013; Yu et al. 2015; Crepin and Rolland 2019). Meanwhile, the sugar signaling of SnRK1 pathway interacts with hormone signaling in a complex regulation, such as ABA, cytokinins and auxin pathways (Rodriguez et al. 2019; Belda-Palazón et al. 2020). Both positive and negative interactions have been reported on SnRK1 and ABA signaling in different (source and sink) organs and conditions (Wurzinger et al. 2018). While ABA leads to stomatal cells closure and photosynthesis repression (Nambara and Marion-Poll 2005; Li et al. 2011; Zhang et al. 2021; Zhou et al. 2022), ZT and IAA promote photosynthesis activity (Li et al. 2019). In addition, CKs antagonize the inhibitory effect of ABA-mediated repression of photosynthesis, through the downregulation of the transcription factor ABI5 (Guan et al. 2014). Compared to CJ03, removing spikelets significantly increased promote hormone metabolism in source leaves of W1844, along with the increased ratio of ABA/ZT and ABA/IAA (Fig. 5). Some recent studies have found that sugar levels interact with CKs to regulate the supply of carbon and energy (Garapati et al. 2015; Sakr et al. 2018). It is more likely that the downregulation of SnRK1 in W1844 is due to the high sucrose status of the leaves, which could be enhanced by the positive effect of CKs and auxin on photosynthetic activity. This observation suggests that W1844 exhibits a significantly higher capacity to regulate sugar status and hormone content, enabling it to control source strength and meet the high sugar demand during the initiation of grain filling (Fig. 6).

## Conclusion

The present study deeply identified the interaction of sink strength and source strength during grain filling initiation. The photosynthetic capacity of W1844 source leaves was higher than that of CJ03, which is partly attributed to its stronger sink strength of inferior spikelets during grain filling initiation. The source leaves of CJ03 and W1844 showed distinct difference in sugar accumulation, SnRK1-related signaling pathway and hormone content, suggesting a potential cross talk of sugar-hormone signaling might be involved in regulating strength of source leaves during grain filling initiation.

### Supplementary Information

**Additional file 1: Fig. S1.** Daily photosynthetically active radiation and daily temperature during the growth period of CJ03 and W1844 at the experiment site of Danyang, Southeast China; The green line indicates a high temperature of 35 °C.** Fig. S2.** Schematic diagram of rice panicle in different treatments; T0, control group with no removing-spikelets; T1, removing top 2/3 of the spikelets in panicle; SS, superior spikelets; IS, inferior spikelets.** Fig. S3.** Grain morphology of different position spikelets in CJ03 and W1844 at 8 DPA in 2020; T0, control group with no removing-spikelets; T1, removing top 2/3 of the spikelets in panicle; SS, superior spikelets; IS, inferior spikelets.** Fig. S4.** Changes in carbohydrates of top three leaves between day and night during early grain filling stage of 2020. T0, control group with no removing-spikelets; T1, removing top 2/3 of the spikelets in panicle; Blue color, carbohydrates accumulation of daytime from 6:00 am to 18:00 am; Orange color, carbohydrates accumulation at 6:00 am (end of night); Grey color, carbohydrates accumulation at 18:00 pm (end of day); Significant differences at each time point with same color are indicated by different letters (*P* < 0.05) as determined by Duncan’s test; The data are the means of three replications ± SD (*n* = 3).**Table S1.** Soil properties of the top soil layer (0–20 cm) before rice planting in 2019 and 2020.** Table S2.** The growth duration of CJ03 and W1844 from 2019 to 2020.** Table S3.** Sequences of primers for Actin and genes for qRT-PCR.

## Data Availability

The datasets generated and/or analysed during the current study are available from the corresponding author on reasonable request.

## Abbreviations

IS: Inferior spikelets
SS: Superior spiklelets
OsSWEET11: *Oryza sativa* Sugar will eventually be exported transporter 11
OsSUTs: *Oryza sativa* Sucrose transporters
SPS: Sucrose-phosphate synthase
SuSase: Sucrose synthase
AGPase: ADP-glucose pyrophosphorylase
T6P: Trehalose-6-phosphate
SnRK1: Snf1-related protein kinase-1
TPS: Trehalose-6-phosphate synthase
TPP: Trehalsoe-6-phosphate phosphatase
ABA: Abscisic acid
CKs: Cytokinins
ZT: Zeatin
IAA: Auxin
SG: Superior spikelets
IG: Inferior spikelets
DPA: Days post anthesis
HPLC–MS/MS: High-performance liquid chromatography–tandem mass spectrometry
ANOVA: Analysis of variance
