# Supplementary material for: The Inferior Grain Filling Initiation Promotes the Source Strength of Rice Leaves
Source: Rice (N Y). 2023 Sep 16;16:41. doi: 10.1186/s12284-023-00656-x (PMC10505135; doi:10.1186/s12284-023-00656-x)
Supplement: Supplementary file 1 — Additional file 1: Fig. S1. Daily photosynthetically active radiation and daily temperature during the growth period of CJ03 and W1844 at the experiment site of Danyang, Southeast China; The green line indicates a high temperature of 35 °C. Fig. S2. Schematic diagram of rice panicle in different treatments; T0, control group with no removing-spikelets; T1, removing top 2/3 of the spikelets in panicle; SS, superior spikelets; IS, inferior spikelets. Fig. S3. Grain morphology of different position spikelets in CJ03 and W1844 at 8 DPA in 2020; T0, control group with no removing-spikelets; T1, removing top 2/3 of the spikelets in panicle; SS, superior spikelets; IS, inferior spikelets. Fig. S4. Changes in carbohydrates of top three leaves between day and night during early grain filling stage of 2020. T0, control group with no removing-spikelets; T1, removing top 2/3 of the spikelets in panicle; Blue color, carbohydrates accumulation of daytime from 6:00 am to 18:00 am; Orange color, carbohydrates accumulation at 6:00 am (end of night); Grey color, carbohydrates accumulation at 18:00 pm (end of day); Significant differences at each time point with same color are indicated by different letters (P < 0.05) as determined by Duncan’s test; The data are the means of three replications ± SD (n = 3).Table S1. Soil properties of the top soil layer (0–20 cm) before rice planting in 2019 and 2020. Table S2. The growth duration of CJ03 and W1844 from 2019 to 2020. Table S3. Sequences of primers for Actin and genes for qRT-PCR. [file 12284_2023_656_MOESM1_ESM.pdf]

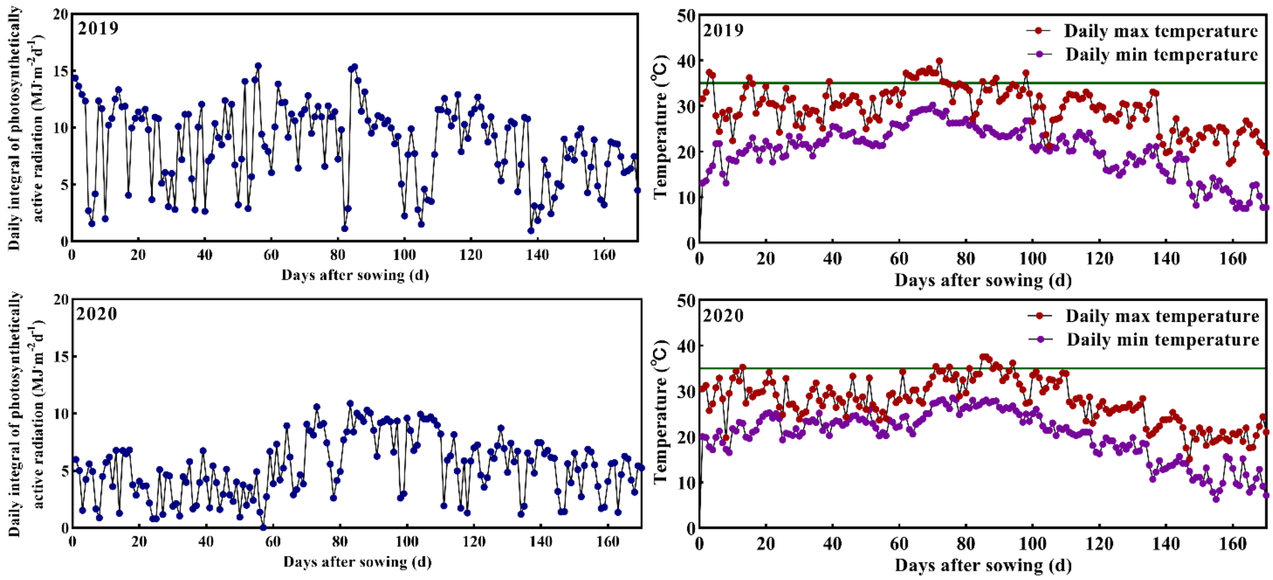

Fig. S1 Daily photosynthetically active radiation and daily temperature during the growth period of CJ03 and W1844 at the experiment site of Danyang, Southeast China; The green line indicates a high temperature of 35◦C

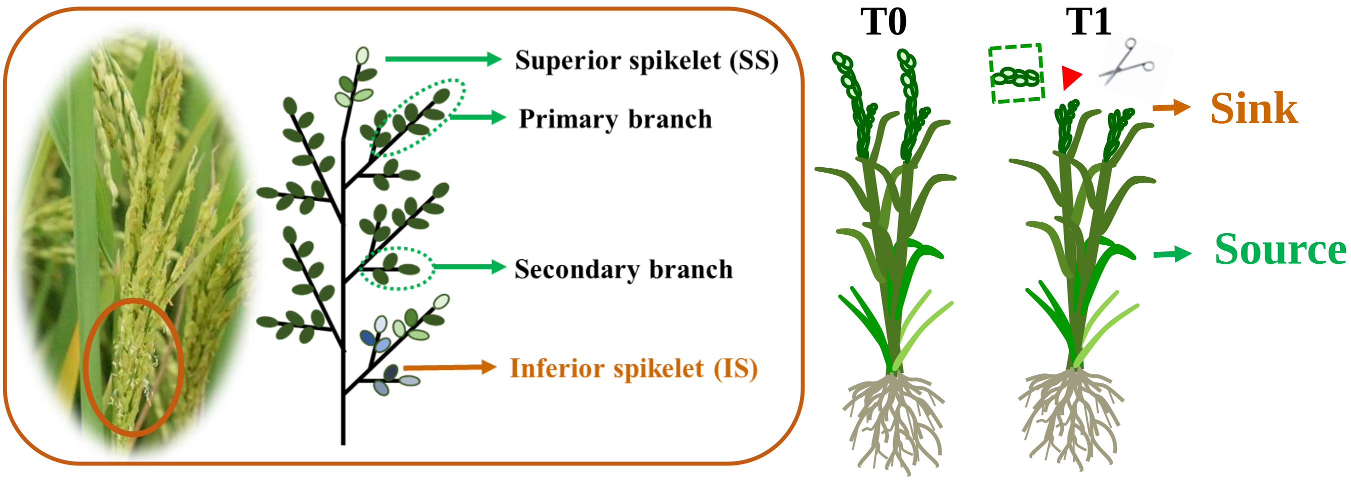

Fig. S2 Schematic diagram of rice panicle in different treatments; T0, control group with no removing-spikelets; T1, removing top 2/3 of the spikelets in panicle; SS, superior spikelets; IS, inferior spikelets

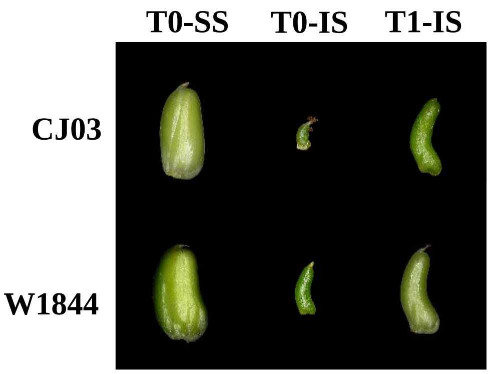

Fig. S3 Grain morphology of different position spikelets in CJ03 and W1844 at 8 DPA in 2020; T0, control group with no removing-spikelets; T1, removing top 2/3 of the spikelets in panicle; SS, superior spikelets; IS, inferior spikelets

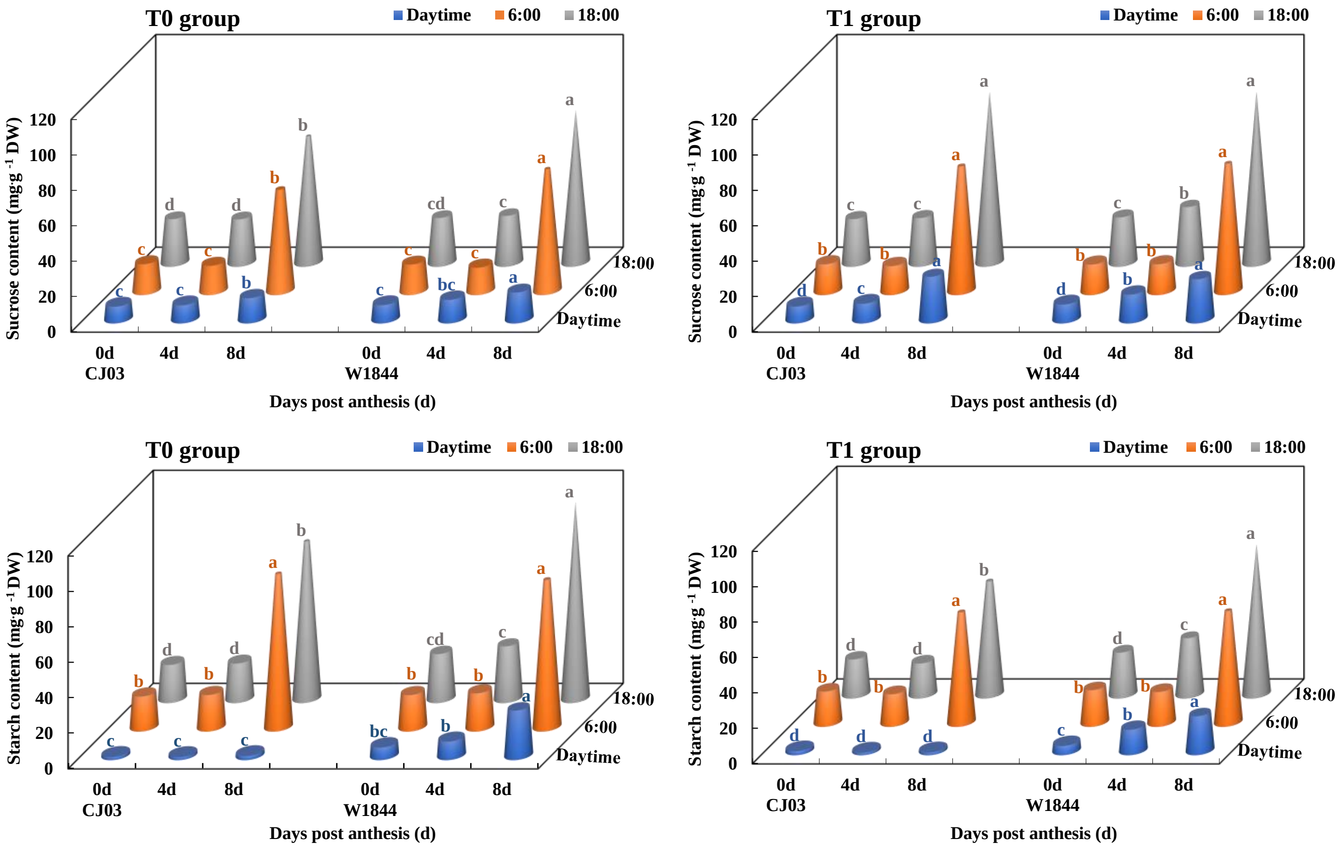

Fig. S4 Changes in carbohydrates of top three leaves between day and night during early grain filling stage of 2020. T0, control group with no removing-spikelets; T1, removing top 2/3 of the spikelets in panicle; Blue color, carbohydrates accumulation of daytime from 6:00 am to 18:00 am; Orange color, carbohydrates accumulation at 6:00 am (end of night); Grey color, carbohydrates accumulation at 18:00 pm (end of day); Significant differences at each time point with same color are indicated by different letters ($P < 0.05$) as determined by Duncan's test; The data are the means of three replications ± SD (n=3)

Table S1. Soil properties of the top soil layer (0–20 cm) before rice planting in 2019 and 2020

Year	PH	Organic matter ($g·kg^{-1}$)	Total N ($g·kg^{-1}$)	NH_4^+-N ($mg·kg^{-1}$)	NO_3^--N ($mg·kg^{-1}$)	Olsens-P ($mg·kg^{-1}$)	NH_4OAc-K ($mg·kg^{-1}$)
2019	6.3	19.5	1.2	6.8	0.9	15.2	138
2020	6.4	19.1	1.3	7.1	0.8	16.9	139.6

Table S2. The growth duration of CJ03 and W1844 from 2019 to 2020

Year	Varity	Heading date (month/day)	Maturity date (month/day)	Duration period from heading to maturity (d)	Total growth duration (d)
2019	CJ03	8/29	11/2	66	165
	W1844	8/31	11/4	66	167
2020	CJ03	9/1	11/3	64	164
	W1844	9/3	11/5	64	166

The duration from heading to maturity and total growth duration are expressed as actual days.

Table S3. Sequences of primers for Actin and genes for qRT-PCR

Gene	TIGR Locus ID	Forward primer 5′ → 3’	Reverse primer 5′ → 3’
Actin	*LOC_Os03g50885*	CAATCGTGAGAAGATGACCC	GTCCATCAGGAAGCTCGTAGC
OsSWEET11	*LOC_Os08g42350*	AAGACCAAGAGCGTCGAGTT	ACGTTCGGGTACATGACGTA
OsSUT1	*LOC_Os03g07480*	GCTTTCAACCAGGGTGTCAG	ACTTTCCGGCACATTGGTTC
OsSUT2	*LOC_Os12g44380*	TCTTTTATCGGTGGGCTGGT	TTGCAAAGAATGGCCGACAA
OsSUT4	*LOC_Os02g58080*	CTCGTGCCCTTTTAGCTGAC	AACGTTTCCAACAGCCATCC
OsSPS1	*LOC_Os06g46450*	CCGACGTGGGTCCATAGAAA	GAAGGCGGCTTGGTTCATTC
OsSUS3	*LOC_Os07g42490*	CCATCGGTTCTCTGCTCTGT	AGCCTTGAGAAGACAGCCAC
OsSUS4	*LOC_Os03g22120*	TCCGTGAACTGGCGAAGACT	CCCAAGTTCGTCACTTGCTG
OsAGPL1	*LOC_Os03g52460*	GGAAAGGTTCCTATTGGAATCG	GGAGGGCTTTATTCCACCTCAG
OsAmy3	*LOC_Os01g51754*	GATCCCGAACGGTGGAAGG	GAACCGGGAGGTCTCATGTC
OsTPS1	*LOC_Os01g23530*	TTGAAGTTCGGTCTGTCG	CTGCCTATCCAAGAACATG
OsTPP2	*LOC_Os10g40555*	ATTGGGTGTTGGTTGTGACC	TTCCTGCATTTGGTTCATCGC
OsTPP6	*LOC_Os08g31630*	CGTGTCCAAGTACCCCAAGG	TAGCTGTCGGGAAATGTGGG
OsOSK1	*LOC_Os11g26910*	AACCAGAGGTAACAGGCAGG	CATCTGTCAAGGAATGCAGG
OsOSK24	*LOC_Os08g37800*	GCAGTGATCCTCATGCCAG	CCTTCGCTGTCTAAGGGACT
OsOSK35	*LOC_Os03g17980*	GCAGTGATCCTCATGCCAG	TAGAACCCTCAGCTTGGTA
OsCYP707A6	*LOC_Os08g36860*	CTACTGCTGATGGTGGCTGA	CCCATGGCCTTTGCTTTAT
OsNCED1	*LOC_Os02g47510*	CTCACCATGAAGTCCATGAGGCTT	GTTCTCGTAGTCTTGGTCTTGGCT
OsABA3	*LOC_Os06g45860*	TGAGATGCTCAAGCTCCAAGT	GCCGACTATTGAGGTCAGAGA
